# Publication rates of congress abstracts is associated with abstract quality: Evaluation of Turkish National Medical Education Congresses and Symposia between 2010 and 2014 using MERSQI

**DOI:** 10.1186/s12909-023-04383-1

**Published:** 2023-05-30

**Authors:** Elif Sarı, Gkionoul Nteli Chatzioglou, Çiğdem Yılmaz Aydın, Ferhat Sarı, Taşkın Tokat, İlke Ali Gürses

**Affiliations:** 1 Department of Ear Nose Throat, School of Medicine, Istanbul Aydın University, Istanbul, Turkey; 2 Department of Anatomy, Faculty of Medicine, Istanbul University, Istanbul, 34093 Turkey; 3 Department of Anatomy, Faculty of Medicine, Istanbul Health and Technology University, Istanbul, Turkey; 4 Department of Public Health, Muğla Provincial Health Directorate, Muğla, Turkey; 5 Department of Pediatric Intensive Care, School of Medicine, Istanbul Aydın University, Istanbul, Turkey; 6 Sakarya University, Education and Research Hospital, Sakarya, Turkey; 7 Department of Anatomy, School of Medicine, Koç University, İstanbul, Turkey

**Keywords:** Medical education, Congresses, Meeting abstract, Peer review, Journal article, Publications, Quality improvement

## Abstract

There are many parameters that could be used to evaluate the quality of scientific meetings such as publication rates of meeting abstracts as full-text articles after the meeting or scoring with validated quality scales/tools that evaluate individual papers, project proposals, or submitted abstracts. This study aimed to determine the full-text publication rates for abstracts presented at Turkish National Medical Education Congresses and Symposia and to assess the quality of given abstracts. Abstracts presented at national medical education congresses and symposia between 2010 and 2014 in Türkiye were evaluated. Initially, the abstracts were evaluated if they were published as full-text articles in international and national peer-reviewed journals following the meeting. Secondly, the quality of presented abstracts was assessed with the Medical Education Research Study Quality Instrument (MERSQI) scale. Overall publication rate for the abstracts was 11.3%. The publication rate of oral and poster presentations were 26.6% and 8.1%, respectively. Oral presentations had a statistically higher publication rate than poster presentations (p = .000). The mean MERSQI score for abstracts was 7.73 ± 2.59. The oral presentations had higher MERSQI mean scores than poster presentations (8.28 ± 2.46 vs. 7.61 ± 2.6; p = .032). Similarly, published abstracts had a significantly higher score compared to unpublished abstracts (10.07 ± 2.74 vs. 7.43 ± 2.41; p = .000). Interestingly, there was no statistical difference between the mean MERSQI scores of the published oral and poster presentations (9.33 ± 2.45 vs. 10.61 ± 2.72; p = .101). This study showed that the main factor for a meeting abstract to be published as a full-text article is the scientific quality of the study. The quality of presentations at annual medical education meetings in Türkiye were low compared with international meetings which did not improve over five years. An institutional policy that would set quality standards for medical education research and increase the awareness of researchers on the topic might help improve the design, execution, and reporting of such studies in Türkiye. The MERSQI could be a valuable tool to monitor the quality of submitted abstracts and to increase the awareness of novice researchers on high quality research.

## Introduction

Scientific meetings provide a venue for physicians, researchers, and other practitioners to discuss the most recent scientific developments in various fields, in which the results of research projects are presented and discussed as oral or poster presentations [1]. Although the main goal of scientific meetings is to share new ideas and experience, which is especially valuable for young researchers, abstracts presented at a congress are expected to be published as full-text articles following a meeting so that the findings of these studies could be considered to be valid, reliable, and beneficial in clinical practice [2]. The publication of a study in an international peer-reviewed journal is a prestigious criterion that both reflects the general quality of the research in question and reveals the scientific level of the congress as well [2].

It is observed that the publication rates of poster and oral presentations in conferences in various fields in Türkiye and abroad vary between 13% and 66% [1, 3–13]. However, the majority of these studies have only examined the rate of publication of the studies presented at the congress in the relevant field as full-text articles [14]. Aside from that, a few detailed papers have reported different aspects such as examining inconsistencies between the congress abstract and the published article possibly resulting from peer-review process and evaluating the factors affecting the final publication of a congress abstract [1, 11, 13, 15, 16]. Additionally, research studies comparing congress abstracts and published full-text articles could reveal ethically questionable practices such as changes in the order and number of authors, author deletions, and reporting inexplicit data in congress abstracts [17, 18].

Assessing the quality of original research studies, meta-analyses, and systematic reviews in clinical and basic medical sciences could be achieved with guidelines and instruments which would provide a valid and reliable instrument to assess the quality of given studies [19–24]. Instruments for evaluating the quality of medical education studies also exist. The Newcastle–Ottawa Scale-Education (NOS-E) was developed to evaluate the quality of non-randomized comparative studies included in a meta-analysis of clinical research and includes three broad domains of (a) selection of the study groups, (b) comparability of the groups, and (c) ascertainment of the outcome of interest [16]. Although content validity of this scale has been established, recent studies reported variable interrater reliability and arbitrary operational definitions [16]. The Medical Education Research Study Quality Instrument (MERSQI) was created as a validated checklist tool in 2007 to assess the quality of experimental, quasi-experimental, and observational studies in medical education [15]. The final MERSQI is an ordinal scoring instrument (with a minimum score of 5 and a maximum score of 18 points) which includes 10 items clustered in six domains: (a) study design, (b) sampling, (c) type of data (subjective or objective), (d) validity of evaluation instrument, (e) data analysis, and (f) outcomes [15]. The instrument demonstrated excellent inter-rater and intra-rater reliability [15, 25]. Similarly, consequent reports have associated higher MERSQI scores with a higher acceptance rate for peer-reviewed journals, higher citation rates, and an increased likelihood of external funding [15, 17, 25]. It is reported that two independent experts are highly correlated with the median quality rating [15].

In Türkiye, the Association for the Advancement of Medical Education (TEGED) (https://teged.org/) organizes annual meetings in the field of medical education. Since 2000, the association has been organizing the National Medical Education Congress which was held biennially. Similarly in 2011, the association also implemented the National Medical Education Symposia that will be held biennially. Thus, organizing annual medical education meetings where researchers and physicians who are interested in the field could share their research.

Although there are numerous publications on the assessment of the quality and publication rates of congress abstracts in several basic and clinical medical fields in our country, no study focused on medical education so far. This study aims to (a) determine the full-text publication rates for abstracts presented at National Medical Education Congresses and Symposia and (b) outline whether the scientific quality of congress abstracts have a role in the full-text publication.

## Materials and methods

### Determination of the sample

The study was carried out between October and December 2019. Initially, the authors tried to obtain the proceedings of the National Medical Education Congresses and Symposia for the abstracts to be evaluated. The proceedings for the National Medical Education Congresses of 2000, 2001, 2006, and 2008 could not be accessed at the official website of the TEGED. The proceedings were available regularly as of 2010. Similarly, the proceedings of all National Medical Education Symposia were available at the official website of the TEGED.

Patel et al. (2011) [9] reported that 98.7% of all eventual publications of meeting abstracts were published within 5 years of presentation at the Congress of Neurological Surgeons and the Congress of American Association of Neurological Surgeons. A similar trend was also reported for Turkish orthopedics and anatomy annual congresses [13, 26].Therefore, the abstracts presented at medical education meetings between 2010 and 2014, i.e. the National Medical Education Congresses held in 2010, 2012, and 2014 and the National Medical Education Symposia held in 2011 and 2013, were included in the study.

### Rates of publication evaluation

For this purpose, possible publications were screened by searching PubMed (2022) and Google Scholar (2022). The entire abstract title, keywords selected from the title and abstract keywords, and surnames of the authors separately or in combinations in English or Turkish were used to identify possible full-text publications [26]. Initially, two authors independently performed article searches and decisions were made unanimously. If two researchers could not agree on an article, the senior author made the final decision.

### Abstract quality evaluation

The MERSQI was used to evaluate all of the abstracts included in the study. MERSQI items were scored on ordinal scales, and the overall MERSQI score was determined by adding the scores. Two authors received training on how to utilize the MERSQI scale before the study. These authors were blinded from the publication data of the abstracts and the scoring was done independently.

### Statistical analysis

The descriptive data of the MERSQI total and subsection scores were calculated after the evaluation of the meeting abstracts using the arithmetic mean ± standard deviation. The conformity of the variables to the normal distribution was examined with visuals (histogram and probability graphs) and analytical methods (Kolmogorov-Smirnov/Shapiro-Wilk tests). The student’s t-test was used to compare the scores in terms of publication status and being oral or poster since there are bivariate categories. One-way ANOVA test was used to compare the total scores by year. In order to find the source of the difference, Tukey test was used in post hoc analysis, and the Bonferroni correction was made. Since the MERSQI is a validated instrument, a separate validation was not performed. The inter-rater reliability was evaluated with Cronbach Alpha Coefficient. The IBM Statistical Package for the Social Sciences, version 25 was used to perform statistical analyses (SPSS Inc; Chicago, IL, USA). The data were given in the 95% confidence interval, and the type-1 error level was used as 0.05 in the analysis of statistical significance.

## Results

**Fig. 1:**
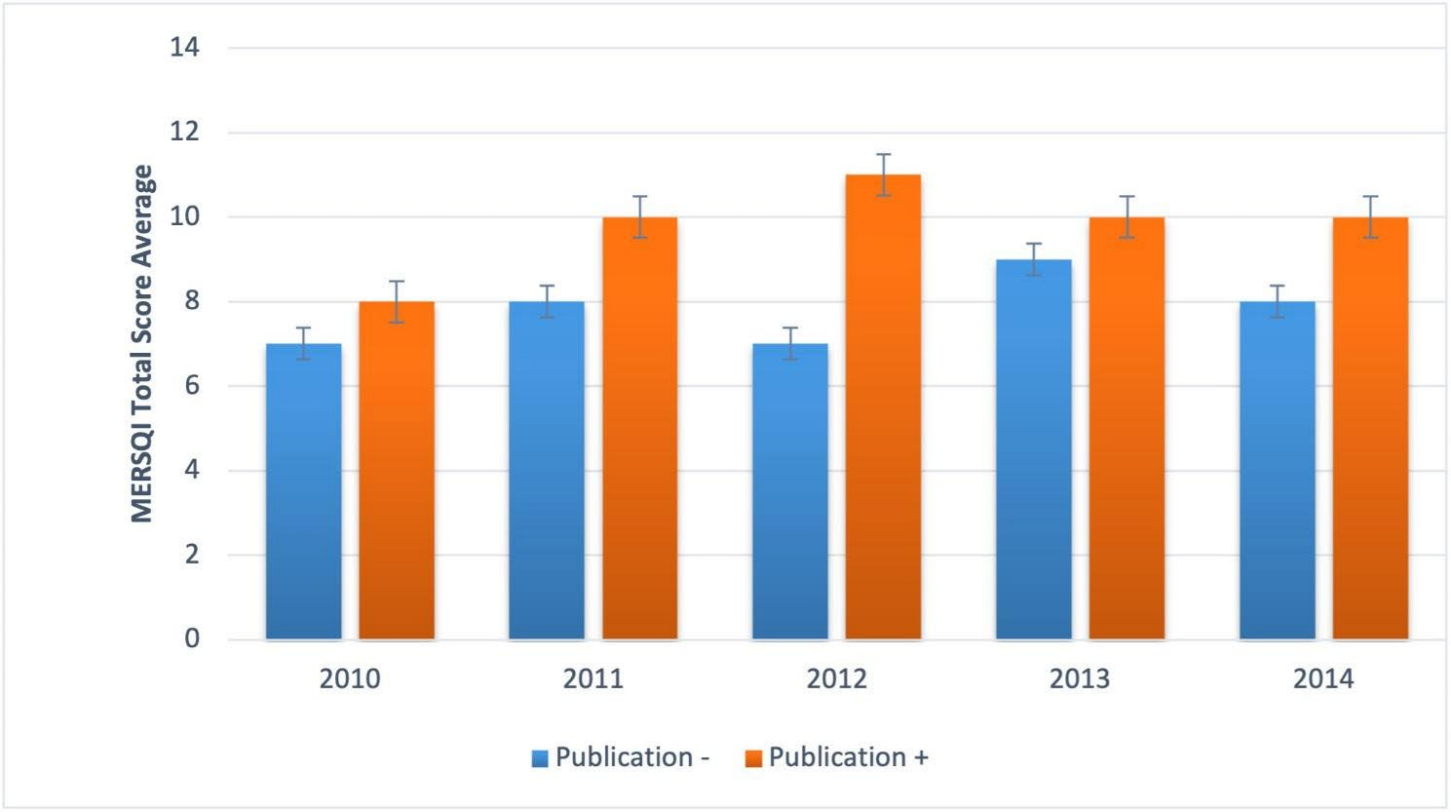
Between 2010 and 2014, the MERSQI Total Score Average of published and non-published abstracts of poster and oral presentations at National Education Congresses and Symposia

**Fig. 2:**
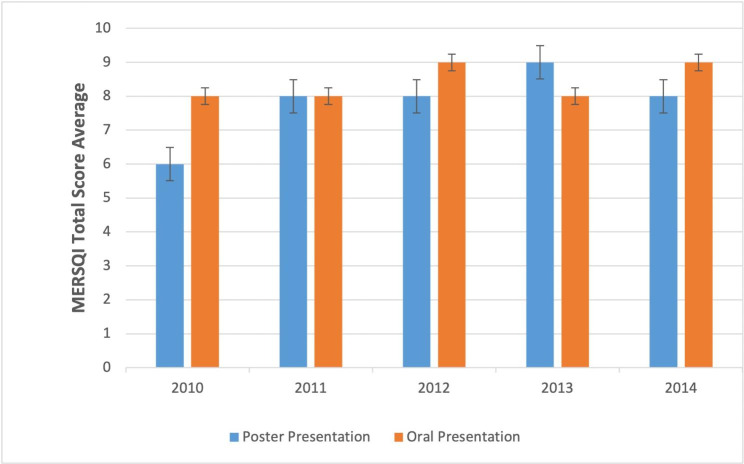
MERSQI total average score of oral and poster presentations presented at National Education Congresses and Symposia between 2010–2014

A total of 449 abstracts were examined. The number of abstracts for National Medical Education Congresses and Symposia were 89 for 2010, 71 for 2011, 113 for 2012, 41 for 2013, and 135 for 2014 (Fig. 1). The total number of oral and poster presentations was 79 (17.5%) and 370 (82.4%), respectively (Fig. 2).

Years were scanned up until June 2019, when the 5-year period required for publishing expired. In international and national peer-reviewed journals, 11.3% (n = 51) of all meeting abstracts were published as full-text articles. The publication rate was 26.6% for oral presentations and 8.1% for poster presentations. In 2010, 6 (24%) of 25 oral presentations, 2 (3%) of 64 poster presentations; In 2011, 2 (11%) of 17 oral presentations, 2 (3%) of 54 poster presentations; In 2012, 6 (60%) out of 10 oral presentations, 9 out of 103 poster presentations (8%); In 2013, 4 (33%) of 12 oral presentations, 4 (13%) of 29 poster presentations; In 2014, 3 (20%) of 15 oral presentations and 13 (10%) of 120 poster presentations were published (Table 1). Oral presentations had a statistically higher publication rate than poster presentations (p = .000). Between 2010 and 2014, the publishing rates were 8.9%, 5.6%, 13.2%, 19.5%, and 11.8%, respectively.

**Table 1:** The number of oral and poster presentations between 2010–2014 and the rates of publication

Year	Oral presentation			Poster presentation			Oral and poster presentations		
	Presentations	Number of publications	Rate of publications (%)	Presentations	Number of publications	Rate of publications (%)	Presentations	Number of publications	Rate of publications (%)
2010	25	6	24	64	2	3	89	8	8
2011	17	2	11	54	2	3	71	4	5
2012	10	6	60	103	9	8	113	15	13
2013	12	4	33	29	4	13	41	8	19
2014	15	3	20	120	13	10	135	16	11

**Fig. 3:**
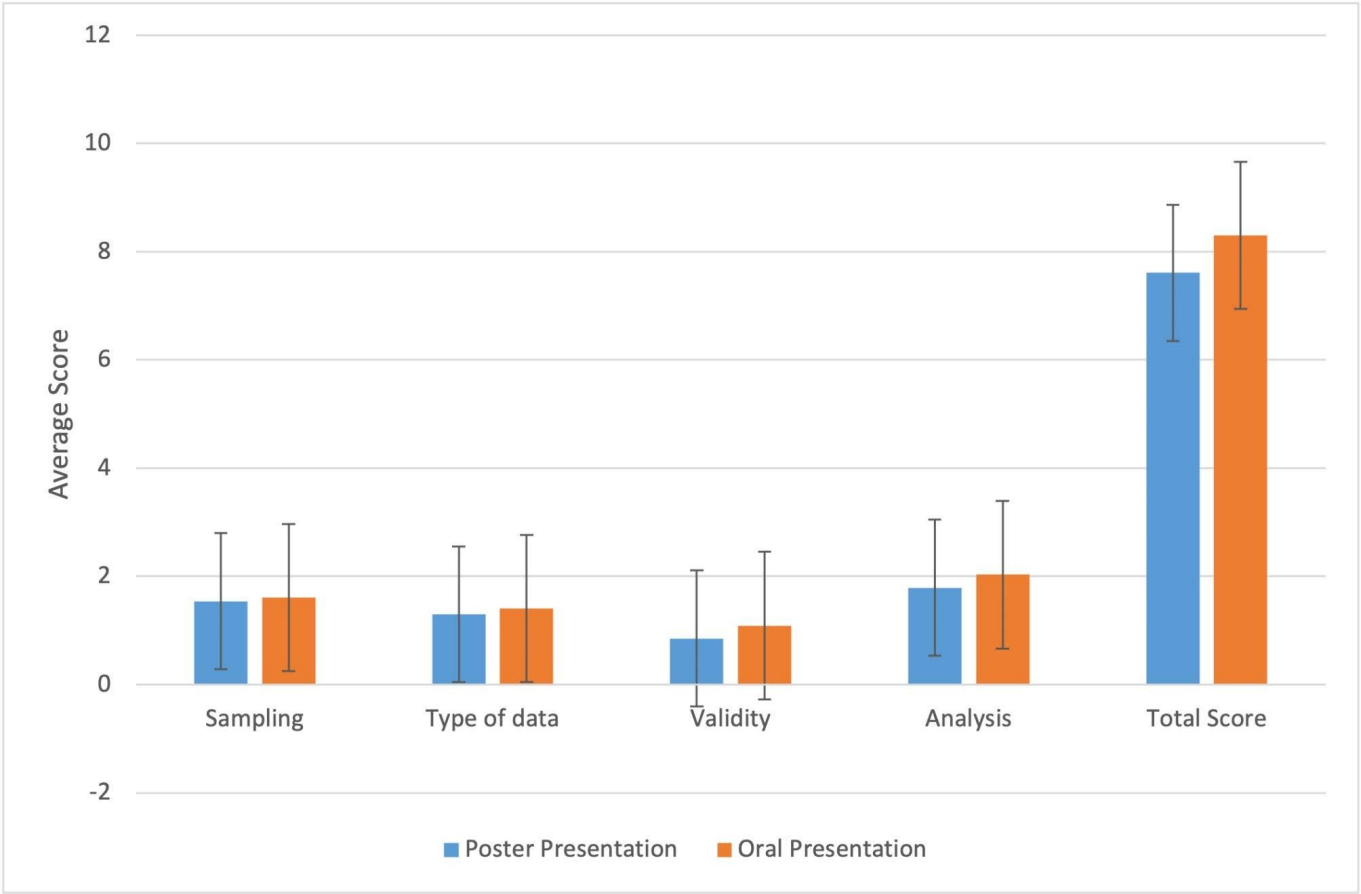
The average scores of MERSQI - subscales scores for poster and oral presentations

The Cronbach Alpha Coefficient for the 10-item MERSQI scale was 0.85. Therefore, one rater’s evaluations were randomly selected for further analysis. Of the reports, 85.9% included single-group cross-sectional studies, 7.5% included pre-post-test design studies, and 5.7% included a comparison group. Randomized trials corresponded to 0.6% of evaluated abstracts. The majority (84.1%) of the studies were conducted in a single institution and 15.8% of them included objective data. In 93.9% of the studies, satisfaction, perception, or opinion outcomes were provided, while knowledge-skill results were examined in only 5.7%. The MERSQI mean-field scores were 0.91 for data analysis, 1.30 for type of data, 0.77 for sampling, 0.29 for validity evidence, and 1.11 for study design. For all abstracts, the lowest MERSQI score was 5 and the highest was 15.5. The mean MERSQI score for all abstracts was 7.73 ± 2.59. The mean MERSQI score for oral and poster presentations were 8.28 ± 2.46 and 7.61 ± 2.6, respectively. Oral presentations received significantly higher mean MERSQI scores (p = .032) compared to poster presentations. Similarly, for MERSQI subscales, oral reports had higher MERSQI scores than poster reports in data analysis (p = .024) and assessment tool validity (p = .031) (Table 2; Fig. 3).

**Table 2:** The mean and the standard deviation of MERSQI and subscales scores for poster and oral presentations between 2010–2014, p < .05 was considered statistically significant

	Oral presentation		Poster presentation		
MERSQI item	Mean	Std. dev.	Mean	Std. dev.	P value
Study design	1.1329	0.29644	1.1068	0.30592	0.488
No. of institutions studied	0.7120	0.40448	0.6081	0.27877	**0.032**
Response rate	0.8987	0.46257	0.9324	0.48504	0.572
Type of data	1.4051	0.80891	1.2973	0.71245	0.275
Internal structure	0.3418	0.47733	0.1608	0.36693	**0.002**
Content	0.5759	0.48759	0.5473	0.49775	0.641
Relationships to the other variables	0.1646	0.37315	0.1405	0.34802	0.583
Appropriateness of data analysis	0.6835	0.46806	0.5122	0.49985	**0.005**
Complexity of analysis	1.3418	0.47733	1.2784	0.44881	0.281
Outcomes	1.0253	0.13631	1,0324	0.12331	0.648
Sampling	1.6108	0.66113	1.5405	0.56283	0.382
Validity of evaluation instrument	1.0823	0.96220	0.8486	0.88960	**0.031**
Data analysis	2.0253	0.81610	1.7905	0.84907	**0.024**
Total score	8.2816	2.46591	7.6162	2.60862	**0.038**

The average MERSQI scores of abstracts between 2010 and 2014 tended to increase annually, with 6.63, 7.84, 7.86, 8.83, and 7.96 respectively. However, this increase was not statistically significant.

**Fig. 4:**
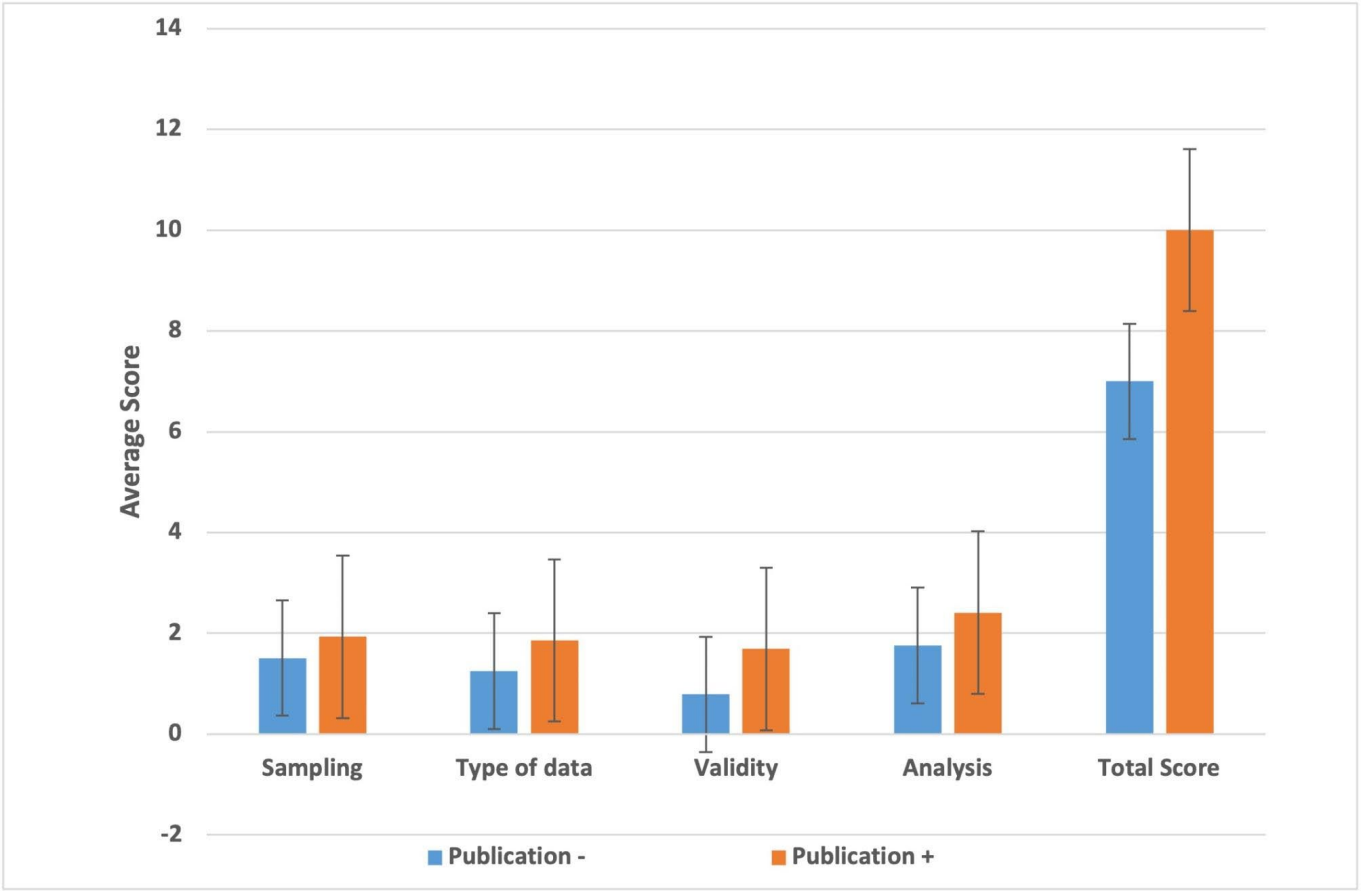
The average scores of MERSQI-subscales scores for published and non-published poster and oral presentations

The mean MERSQI scores for abstracts followed by a full-text article was significantly higher compared to abstracts that were not published (10.07 ± 2.74 vs. 7.43 ± 2.41; p = .000). The abstracts that were published also received higher MERSQI scores for subscales of type of data (p = .000), sampling (p = .000), the validity of evaluation instrument (p = .000), and data analysis (p = .000) than those that were not published (Table 3; Fig. 4). The mean MERSQI scores of the published oral and poster presentations were 9.33 ± 2.45 and 10.61 ± 2.72, respectively. This difference did not reach statistical significance (p = .101).

**Table 3:** The mean and the standard deviation of MERSQI and subscales scores for published and non-published poster and oral presentations between 2010–2014, p < .05 was considered statistically significant

	Publication +		Publication -		
MERSQI item	Mean	Std. dev.	Mean	Std. dev.	P value
Study design	1.1667	0.36968	1.1043	0.29446	0.257
No. of institutions studied	0.7843	0.43880	0.6062	0.27979	**0.007**
Response rate	1.1373	0.45911	0.8995	0.47740	**0.001**
Type of data	1.8627	1.00039	1.2462	0.65797	**0.000**
Internal structure	0.5098	0.50488	0.1520	0.35861	**0.000**
Content	0.8137	0.38679	0.5188	0.49838	**0.000**
Relationship to other variables	0.3529	0.48264	0.1181	0.32312	**0.001**
Appropriateness of data analysis	0.8235	0.38501	0.5063	0.49996	**0.000**
Complexity of analysis	1.5882	0.49705	1.2513	0.43428	**0.000**
Outcomes	1.0392	0.13576	1.0302	0.12434	0.628
Sampling	1.9216	0.59475	1.9216	0.59475	**0.000**
Validity of evaluation instrument	1.6765	1.03838	0.7889	0.83722	**0.000**
Data analysis	2.4118	0.77914	1.7575	0.82751	**0.000**
Total score	10.0784	2.74840	7.4328	2.41741	**0.000**

## Discussion

This study evaluated the publication rates of abstracts presented at Turkish Medical Education Congresses and Symposia for the first time and showed that abstracts with higher MERSQI scores were followed by a full-text article more frequently. Between 2010 and 2014, 11.3% of abstracts presented at the National Medical Education Congress and Symposia were published as full-text articles in national and international peer-reviewed journals. There are studies showing that the publication rates of international medical education meeting abstracts are 35% and 44% [17, 25]. In our study, this rate was shown to be fairly low when compared to international meetings reported in the literature. Design of a study is an identified reason for higher publication rates of congress abstracts. For example, in different medical congresses, studies with randomized controlled design and experimental studies tend to be published more frequently compared to case presentations [7, 10, 13, 26, 27]. Similarly, the majority (85.9%) of the studies presented at National Medical Education Congresses and Symposia were single-group cross sectional studies and only 0.6% were randomized trials. This might give a general idea that the design of abstracts presented at Turkish Medical Education meetings is one of the reasons for the reported low publication rate.

Literature shows that oral presentations are more frequently published as full-text articles both in various clinical and basic medical fields (4.3–68.4% for oral presentations vs. 1.3–28.1% for poster presentations) [9, 10, 12–14, 26–29]. Our study shows a similar trend of a publication rate of 26.6% for oral presentations and 8.1% for poster presentations. This might be because better-designed and higher-quality studies with higher scientific value get accepted as oral presentations at congresses.

Apart from study design, factors such as sampling of participants, using a validated evaluation instrument, and appropriate data analysis methods are important for evaluation of the quality of a given educational study. In that context, the mean MERSQI scores for medical education research were reported to be ranging from 9.05 to 9.95 [15, 17, 25, 30, 31] and the abstracts having a MERSQI score of 10 or higher have been demonstrated to be more likely to be published [25]. Our study showed that the mean MERSQI score for abstracts presented at the National Medical Education meetings in Türkiye was 7.73, which was lower than previous studies in the literature. While the MERSQI mean score of the unpublished abstracts was 7.43 ± 2.41, the mean score for the published papers was 10.07 ± 2.74. It is expected that paying more attention to quality measures including sampling, validity of evaluation instruments, data analysis, and clear outcomes during the planning or evaluation of medical education research may enhance the possibility of full-text publication.

Similar to publication rates, the mean MERSQI score for oral presentations were significantly higher when compared to poster presentations (8.28 ± 2.46 vs. 7.61 ± 2.6; p = .032). Additionally, evaluation of the MERSQI tool revealed that the oral presentations outperformed poster presentations in data analysis and validity of evaluation instrument as well. These findings supported the notion that better-designed and higher-quality studies get to be accepted as oral presentations at congresses. Nevertheless, our study did not reveal a difference between oral and poster presentations for other domains such as study design, sampling, and outcomes.

Interestingly, comparison of MERSQI scores for oral and poster presentations that were published as full-text articles showed no statistical difference (9.33 ± 2.45 vs. 10.61 ± 2.72). Previously, Smith et al. (2017) [30] reported that there was no difference in methodological quality between oral and poster presentations. This finding suggests that despite poster presentations being less frequently published and receiving lower MERSQI scores, high quality poster presentations have a similar chance of full-text publication following a meeting.

Finally, our study showed that despite the total publication rate and mean MERSQI scores for the abstracts presented at National Medical Education Congresses and Symposia increased over the years, this increase was not statistically significant. Five consecutive meetings might not be adequate to assess the change in publication rates or quality of the abstracts, however, it might also show either a lack of awareness in researchers and organizers regarding the MERSQI tool for quality control of submitted abstracts or a lack of institutional policy for setting a quality standard. Although the authors did not come across any quality standards requested of researchers at the official websites for the meetings, a longitudinal quality analysis would reveal any implicit policy that resulted in the increase of quality of submitted abstracts.

Our study has a few limitations. First of all, the evaluated abstracts were a decade old. Therefore, we do not know if the abstracts submitted to current meetings have a higher MERSQI score or not. Although it would not be possible to evaluate publication rates for the meetings within the last few years, a longitudinal study that evaluates all available abstracts might reveal any existing change in the quality. Secondly, this study did not evaluate whether the researchers who submitted the abstracts, the scientific committee members of given meetings, or the TEGED were aware of the MERSQI tool and are willing to use this tool in designing and reporting their studies. A survey study with the authors of published and unpublished abstracts, along with the association might reveal the awareness of the researchers and organizers. Finally, since the MERSQI tool was already validated, an additional validation was not designed during this study. Similarly, intra-rater reliability was not performed. Therefore, existing results were based on inter-rater reliability only.

## Conclusion

The authors acknowledge that passing peer-review and publication as a full-text article can be a quality indication for a congress abstract, and analyzing publication rates of scientific congresses might help us to monitor a meeting’s scientific quality over time. It should also be noted that publishing is not a race and the main goal of scientific meetings is to share new ideas and experience. This kind of interaction is especially valuable for students and novice researchers. In this regard, trying to increase the quality of studies presented at scientific meetings might reinforce this experience, especially if this goal is achieved without creating a pressure for peer-reviewed publication. One way to obtain this goal is setting an institutional policy on study quality for submitted abstracts. It seems that an institutional policy on improving the quality of medical education research in Türkiye is needed. The MERSQI tool could be implemented as quality standards or used for the quality assessment of abstracts submitted to national medical education congresses and symposia in Türkiye. Once a policy is set, announcement of this practice would increase the awareness of researchers who attend given meetings. Familiarizing young researchers with an existing quality tool would help them to improve the design, execution, and evaluation of future medical education research projects.

## Data Availability

Data are available from the Corresponding author upon reasonable request.
